# A five-gene signature predicts overall survival of patients with papillary renal cell carcinoma

**DOI:** 10.1371/journal.pone.0211491

**Published:** 2019-03-01

**Authors:** Ze Gao, Dong Zhang, Yi Duan, Lei Yan, Yidong Fan, Zhiqing Fang, Zhaoxu Liu

**Affiliations:** 1 Department of Urology, Qilu Hospital of Shandong University, Jinan, Shandong, China; 2 Department of Breast Surgery, Qilu Hospital of Shandong University, Jinan, Shandong, China; 3 School of Nursing, Shandong University, Jinan, Shandong, China; 4 Department of Oncology and Pathology, Karolinska Institutet, Stockholm, Sweden; University of South Alabama Mitchell Cancer Institute, UNITED STATES

## Abstract

**Background:**

The present study aims to investigate the gene expression changes in papillary renal cell carcinoma(pRCC) and screen several genes and associated pathways of papillary renal cell carcinoma progression.

**Methods:**

The papillary renal cell carcinoma RNA sequencing (RNA-seq) data set was downloaded from TCGA (The Cancer Genome Atlas). We identified the differentially expressed mRNAs between cancer and normal tissues and performed annotation of differentially expressed mRNAs to figure out the functions and pathways they were enriched in. Then, we constructed a risk score that relied on the 5-mRNA. The optimal value for the patients’classification risk level was identified by ROC analysis. The relationship between mRNA expression and prognosis of papillary renal cell carcinoma was evaluated by univariate Cox regression model. The 5-mRNA based risk score was validated in both complete set and testing set.

**Result:**

In general, the 5-mRNA (CCNB2, IGF2BP3, KIF18A, PTTG1, and BUB1) were identified and validated, which can predict papillary renal cell carcinoma patient survival. This study revealed the 5-mRNA expression profile and the potential function of a single mRNA as a prognostic target for papillary renal cell carcinoma.

**Conclusion:**

In addition, these findings may have significant implications for potential treatments options and prognosis for patients with papillary renal cell carcinoma.

## 1.Introduction

The incidence of kidney cancer approximately accounted for 2.4% of adult malignancies, estimated 338,000 new cases in 2012[1]. Renal cell carcinoma (RCC), the seventh most common histological type of cancer, affects men more frequently than women and comprises nearly 90% of all kidney tumors[2, 3], with clear cell (70%), papillary (10–15%), and chromophobe (5%) carcinoma the main histologic types[4]. Papillary renal cell carcinoma (pRCC) is a renal parenchyma malignant tumor with papillary or tubulopapillary architecture, including type 1 and type 2 pRCC[5]. According to the original description, type 1 is often multifocal, characterized by single layered small cell and scanty cytoplasm whereas type 2 is heterogeneous, characterized by large pseudostratified cells and eosinophilic cytoplasm[6]. The division of pRCCs into type 1 and type 2 tumors has been proved to have prognostic significance. The mutation of MET oncogene is a crucial step into the pathogenesis of hereditary pRCC forms, resulting in constitutive activation of the tyrosine kinase domain, which leads to increasing unregulated proliferation, invasion and metastases. However, they can be found only in a small proportion of sporadic cases[7] and a greater number show somatic copy number gains involving chromosome 7q[8, 9]. Although papillary renal cell carcinoma is indolent, bilateral and multifocal in some patients, other patients have a separate lesion with an aggressive clinical course[10]. The pRCC type 1 has been associated with overexpression or activation mutation of MET proto-oncogene which encodes for a hepatocyte growth factor receptor (HGFR)[11]. The pRCC type 2 is concerned with activation of the NRF2/antioxidant response element (ARE) pathway[12]. It has been reported that the 5-year overall survival of localized pRCC rates of 78–79% and cancer-specific 5-year survival rates of 86–94%[13, 14]. Unfortunately, there are currently no effective forms of therapy for patients with advanced disease. Although many patients have greatly different treatment responses and prognoses, their tumor types are identical in histology.

With the rise of gene sequencing, a large number of messenger RNAs (mRNA) have been discovered in the role of cancer. The mRNA is a large family of RNA molecules that convey genetic information from DNA to the ribosome, where they specify the amino acid sequence of the protein products of gene expression. A lot of evidences show that mRNAs are wildly involved in fundamental cellular processes, such as cell differentiation, proliferation, growth, mobility, and apoptosis, as well as carcinogenesis or cancer progression.

Recently, large-scale gene data sets regarding pRCC were downloaded from TCGA database (https://cancergenome.nih.gov/). The R Programming Language (R) was utilized for preprocessing and analysis of these data to obtain the differentially expressed genes (DEGs). In the present study, we screened the differentially expressed mRNAs between pRCC tissues and matched normal tissues, and found the association of different mRNAs expression with clinical data. We developed and validated a risk score for a prognosis based mRNA signature to demonstrate the relationship between mRNA and the prognosis of pRCC by using sample segmentation and Cox regression analysis.

## 2.Materials and methods

### 2.1 Data source and data pre-processing

The RNA sequencing data with papillary renal cell carcinoma (pRCC) was downloaded from TCGA dataset (June 13.2018). There were 321 pRCC samples in total, including 289 primary pRCC and 32 normal ones. In total, we screened 285 available samples from 321 samples, 1 sample information was repeated, and 3 samples lost survival data. The inclusion criteria were as follows:(1) a histological diagnosis of papillary renal cell carcinoma, which is a subtype of renal cell carcinoma, (2) patients underwent radical nephrectomy or partial nephrectomy, (3) patients that didn’t receive any neoadjuvant treatment, (4) patients with corresponding RNA-seq data, (5) patients with explicit clinical prognostic data. Besides, the corresponding clinical characteristics, including sex, age, pathological stage, clinical stage, definite TNM stage and histological type of our cohort were integrated as a clinical statistical chart in Table 1. Signal value for all genes were transformed to the log base 2. Standardization of the level of mRNA expression for each sample was normalized with median quantification. Since the data comes from the TCGA database, no further approval is required from the Ethics Committee.

**Table 1:** Clinical parameters of patients in the papillary renal cell carcinoma cohort obtained from The Cancer Genome Atlas (n = 285).

Parameter	Value	Hazard ratio	95%CI	P value
**Age(years)**	61(28–88)	1.004	0.997–1.031	0.792
**Sex**				0.149
**Female**	76	1.000		
**Male**	209	0.617	0.320–1.190	
**Pathologic stage**^**a**^				2.0E-14
**StageⅠ**	170	1.000		
**StageⅡ**	21	0.958	0.214–4.295	
**StageⅢ**	50	4.779	2.260–10.108	
**StageⅣ**	15	16.112	6.698–38.758	
**Clinical stage**^**b**^				
**StageⅠ**	138	1.000		<2.0E-16
**StageⅡ**	21	0.570	0.074–4.423	
**StageⅢ**	29	6.561	2.891–14.892	
**StageⅣ**	10	25.639	9.679–67.920	
**Tumor type**^**c**^				
**Type 1**	76	1.000		0.100
**Type 2**	84	2.769	1.013–7.571	
**T stage** ^**d**^				
**T1**	191	1.000		2.0E-08
**T2**	32	3.161	1.307–7.645	
**T3&T4**	60	6.815	3.408–13.66	
**N stage**^**e**^				1.0E-11
**N0**	144	1.000		
**N1&N2**	28	7.715	3.864–15.400	
**M stage** ^**f**^				<2.0E-16
**M0**	206	1.000		
**M1**	11	45.660	15.560–134.0	

^a^Pathologic stage was not available in 29 case

^b^clinical stage was not available in 87 case

^c^Tumor type was not available in 124 case

^d^T stage was not available in 2 case

^e^N stage was not available in 113 case

^f^ M stage was not available in 68 case.

Age is reported as the median(range).

### 2.2 Screening of differentially expressed mRNA

The RNA sequencing data (level 3) of 321 pRCC samples was downloaded from the TCGA data portal. The differentially expressed mRNAs between pRCC tissues and matched normal ones were analyzed using the limma package in R[15]. The edgeR package and DESeq2 package in R were subsequently used for the calculation of DEGs. The adjust *P* value <0.05 and |log_2_FC|>1 were set as the cut-off criteria. The genes that presented in both edgeR and DESeq2 package analysis results were selected as the final DEGs.

In our study, we mainly used the program code written in R language to analyze and deal with RNA-seq data.

### 2.3 Identification and selection of prognostic related mRNA

The patients were further randomly divided into the training set (n = 143) and the testing set (n = 142) with caret package in R language. In the training set, the association between mRNAs expression and the overall survival time of patients were analyzed. Univariate Cox regression analysis was performed with Survival package in R language[16]. In the survival analysis, We selected the time that patient's initial pathological diagnosis to papillary renal cell carcinoma as the start point, and the time that death or censored events occurred as the end point. Messenger RNAs with expressing significance *p* values <0.05 were selected as a target mRNA. To further ensure the reliability and feasibility of clinical prognosis based on mRNA, we made a Robust likelihood-based survival analysis by using Rbsurv package in R[17, 18]. All 285 samples were again randomly assigned to a training set or a testing set with the caret package in R language. We fitted a gene to the training set of samples and obtained a corresponding parameter estimate. Then we evaluated log likelihood with the parameter estimate and the testing set of samples. This procedure was repeated 10 times independently, which resulted in 10 log-likelihood for each gene. The best gene, g (1), with the largest mean log likelihood was selected. We searched the next best gene by evaluating every two-gene model and selected an optimal one with the largest mean log likelihood. Akaike information criterions (AICs) for all the candidate models were computed and an optimal predictive model was selected as with the lowest AIC value. The above procedure was repeated 1000 times, thus obtained 1000 loglike*s for each gene. The mRNA with the highest frequency (freq>300) was selected as the final target mRNA.

### 2.4 Establishment and validation risk score formula

The association between DEGs and patients’overall survival time was analysed in training set. Univariate Cox regression analysis was performed in R language by Survival package with the threshold of *p* value set as 0.05. In the univariate Cox regression analysis of the training set, these prognostic-related mRNAs were included in their estimated regression coefficients, and a risk scoring formula was established. In the training set, the patients were divided into high-risk score or low-risk score group based on the optimal risk score cutoff value, which represented the point at which the Youden index (sensitivity + specificity– 1) reached a maximum value in training set. Then, we applied the optimal risk score cutoff value to the validation set and the whole pRCC cohort to evaluate the classification performance of the model. By using survival ROC package in R, the receiver operating characteristic (ROC) curve was obtained. Then, choosing the optimal cutoff point with maximum sensitivity and specificity. Based on the optimal cut-off value, the survival difference between the low-risk and high-risk group was evaluated. The accuracy of the risk score formula was then further verified in the testing set and the complete set.

### 2.5 Functional and pathway enrichment analysis of DEGs

The ConsensusPathDB (http://cpdb.molgen.mpg.de/) database is a molecular functional interaction database, integrating information on protein interactions, genetic interactions signaling, metabolism, gene regulation, and drug-target interactions in humans. In order to better understand the biological functions and characteristic, the present study performed Gene ontology (GO) using clusterProfiler package in R and Kyoto Encyclopedia of Genes and Genomes (KEGG) pathway analyses in ConsensusPathDB database. All genes from human beings were used as reference. In this study, we analyzed the DEGs that were significantly up- and down-regulated as determined from pRCC data. The p value of the univariate survival analysis was less than 0.05 and considered statistically significant. To correct errors following multiple comparison analysis, the Benjamini-Hochberg step-up method was used to control the FDR.

## 3.Result

### 3.1 Differentially expressed mRNAs in pRCC

**Fig 1:**
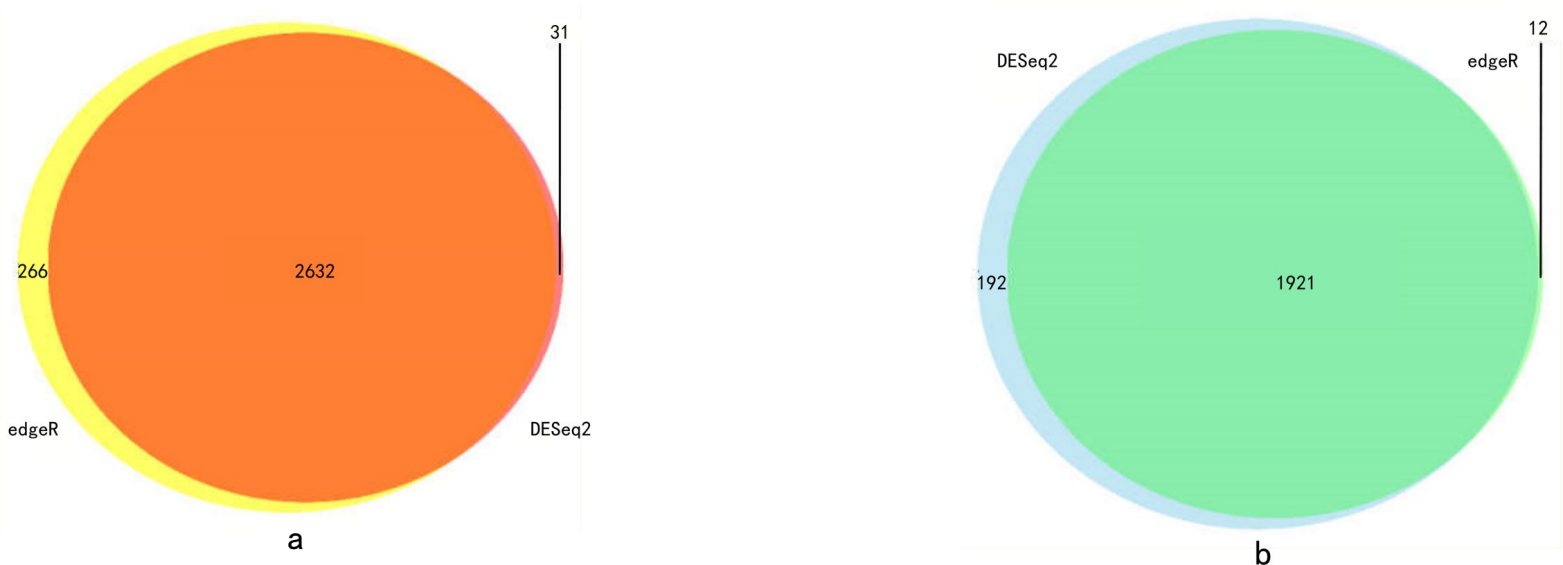
(a)The Venn polt of up-regulated DEG. (b)The Venn polt of down-regulated DEG.

**Fig 2:**
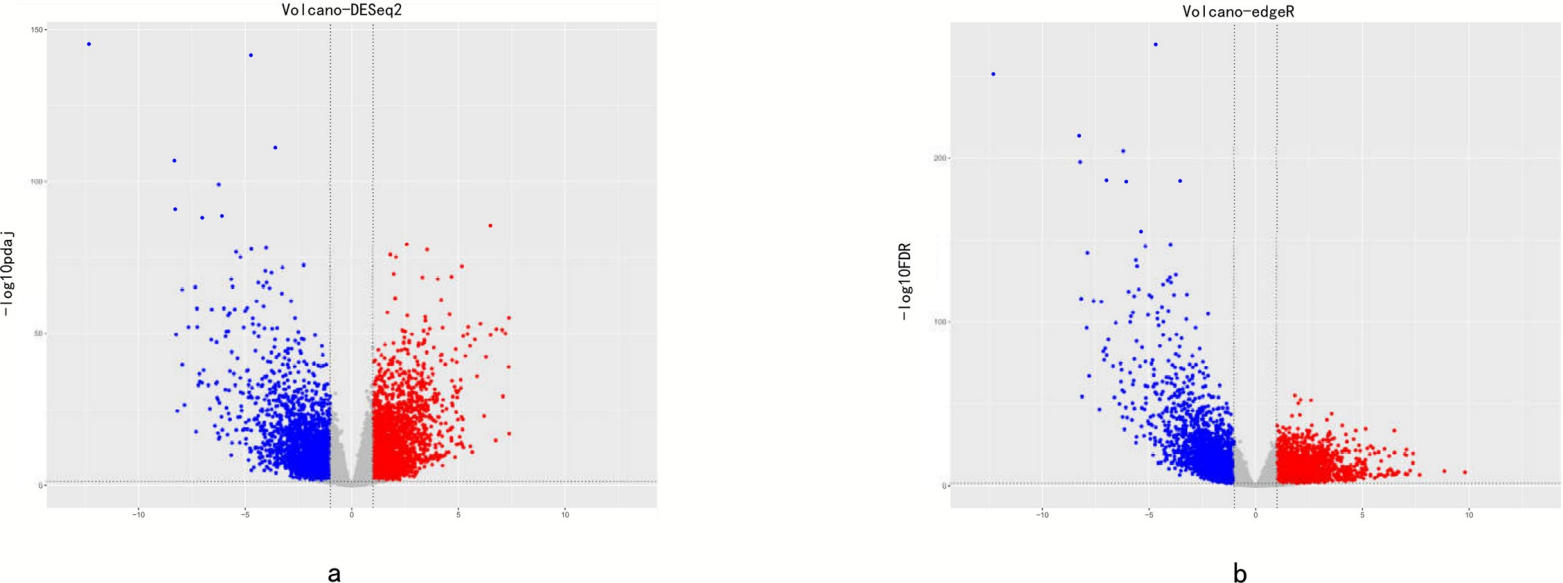
Volcano map with edgeR and DESeq2.

With a cut-off value of *P* < 0.05 and |log2FC| >1.0, 4776 and 4831 differentially expressed mRNAs were identified by using the DESeq2 and edgeR package in R language, respectively (S1 File). The genes that presented in both edgeR and DESeq2 package analysis results were selected as the final DEGs. According to this standard, 2632 up-regulated mRNAs and 1921 down-regulaed ones were identified (Fig 1A and 1B). We displayed the distribution of all the differentially expressed mRNAs in both the–log(FDR) and logFC dimensions through a volcano plot in Fig 2.

### 3.2 Identification and selection of prognostic related mRNA

**Fig 3:**
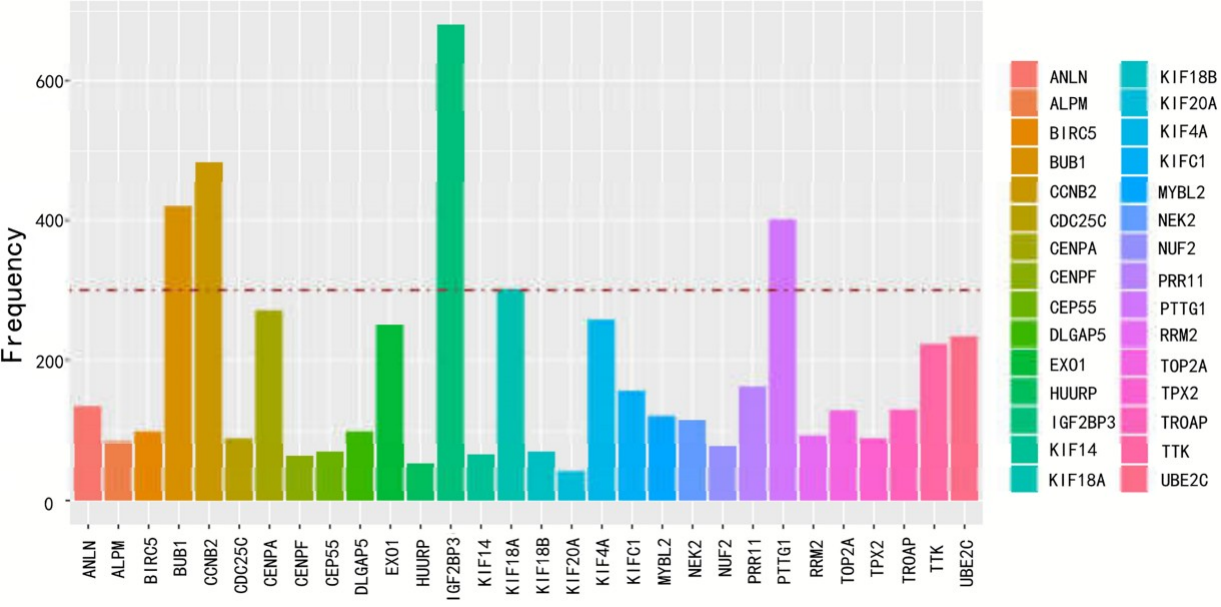
Statistic frequency analysis of the significantly altered mRNAs.

The total of 4553 mRNAs was further randomized into a training set and a testing set. A total of 749 differential expressed mRNAs were identified by univariable cox regression with the *P* <0.05 in training set (S2 File). The top of 20 genes with the lowest *p* value were selected and presented in Table 2. Random data analysis was performed by using Robust likelihood-based modelling for 1000 times. Statistic frequency analysis of the significantly altered mRNAs indicated that all the selected mRNAs had a high frequency (Fig 3). In further analysis, differentially expressed mRNA with a frequency above 300 was picked out. Finally, there were 5 mRNAs identified as prognostic feature (Table 3). Among these genes, four mRNAs (CCNB2, IGF2BP3, KIF18A, PTTG1) had positive coefficients which suggested that higher expression level was associated with worse survival time and one (BUB1) had negative coefficients suggested that higher levels of expression were related with better survival time.

**Table 2:** Top 20 mRNAs significantly associated with the survival time of patients in the training set(n = 143).

mRNA	HR	Cox *p* value	CI 95
IGF2BP3	1.72	0	1.42–2.09
CENPA	2.18	1.00E-06	1.6–2.97
CCNB2	2.2	1.00E-06	1.6–3.02
KIF18A	2.69	2.00E-06	1.78–4.06
CLDN11	1.66	7.00E-06	1.33–2.08
LOC101928583	1.66	7.00E-06	1.33–2.08
TTK	1.97	7.00E-06	1.47–2.65
ZNF488	1.67	1.10E-05	1.33–2.09
KIF20A	1.92	1.20E-05	1.43–2.57
DEPDC1	1.65	1.20E-05	1.32–2.06
BUB1	1.93	1.20E-05	1.44–2.6
PTTG1	2.26	1.30E-05	1.57–3.27
ASPM	1.65	1.50E-05	1.32–2.07
NEK2	1.73	1.50E-05	1.35–2.21
CEP55	1.8	1.70E-05	1.38–2.35
TOP2A	1.7	2.00E-05	1.33–2.16
TPX2	1.94	2.20E-05	1.43–2.63
NUF2	1.8	2.50E-05	1.37–2.36
SOX11	1.56	2.60E-05	1.27–1.91
DLGAP5	1.76	2.80E-05	1.35–2.29

**Table 3:** Significantly altered mRNAs with frequency above 300.

mRNA	Count
BUB1	421
CCNB2	484
IGF2BP3	681
KIF18A	302
PTTG1	401

### 3.3 The 5-mRNA signature predicts the survival of patients with pRCC

**Fig 4:**
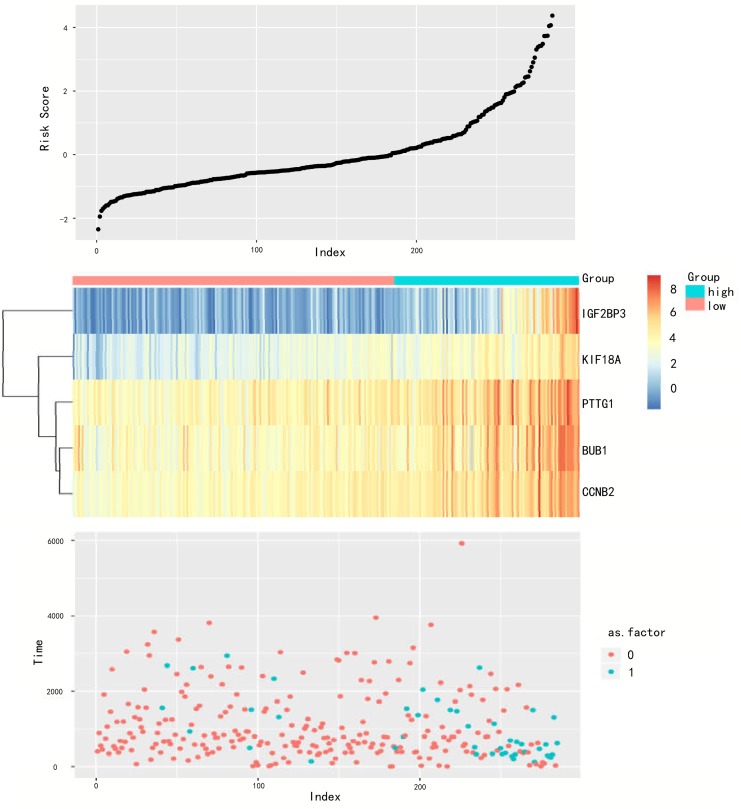
mRNA risk score analysis of the training set. The mRNA signature risk score distribution heat-map of the mRNA expression profiles.Rows represent mRNAs, and columns represent patients.

A risk score formula based on the expression level and coefficient of 5 mRNAs was created as follows: Risk score = (-0.560 * expression level of BUB1) + (0.337 * expression level of CCNB2) + (0.424 * expression level of IGF2BP3) + (0.516 * expression level of KIF18A) + (0.310 × expression level of PTTG1). Then, the mRNAs signature based risk score for each patient in training set were calculated, and patients in the cohort were assigned into high-risk group (n = 51) and low risk group (n = 92). The risk score was inversely related to the patients’survival time. The patients with lower risk score had longer survival time and death patients had higher risk score (Fig 4). The patients in high risk group had a tendency to have higher expression levels of IGF2BP3, KIF18A, PTTG1, BUB1 and CCNB2.

### 3.4 Available prognostic indicator in pRCC patients

**Fig 5:**
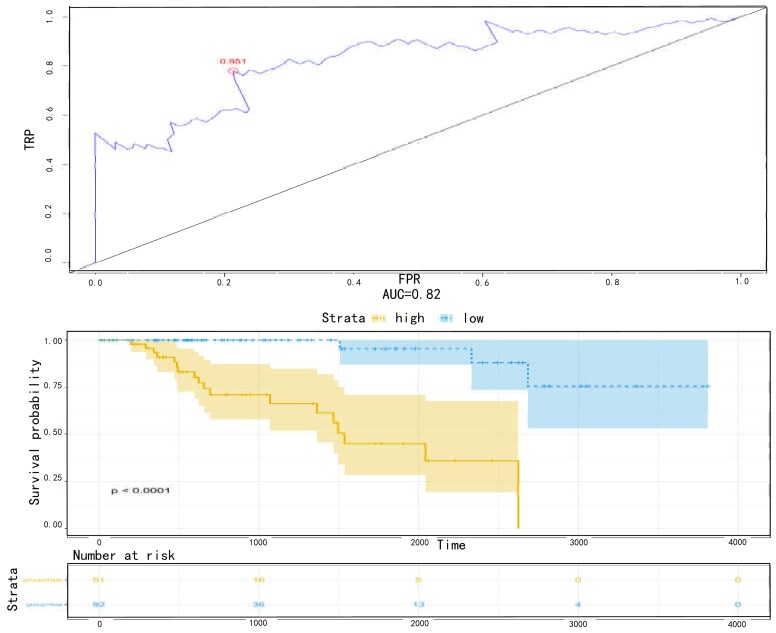
(a) ROC for 5 mRNA,Method = KM. Receiver operating characteristic (ROC) analyzes the sensitivity and specificity of the survival time by risk score based on the 5-mRNA signature. The red dot indicates the optimal cut-off point. (b) Method Kaplan Meier. Kaplan-Meier estimated the survival time of the training set patients using risk score based on the 5 mRNA signature. The plot was used to visualize the survival probability for the low-risk versus high-risk group of patients based on the optimal cut-off point.

We performed ROC analysis for the 5-mRNA risk score in the training set to further validate the sensitivity and specificity of survival prediction. The optimal cut-off value for false negative and false positive minimum was 0.951, and the area under the curve (AUC) was 0.82 (Fig 5A). The Kaplan-Meier curves showed that patients in the high-risk group had significantly shorter overall survival than those in the low-risk group (log-rank test p < 0.0001)(Fig 5B).

### 3.5 External validation of five-gene signature

**Fig 6:**
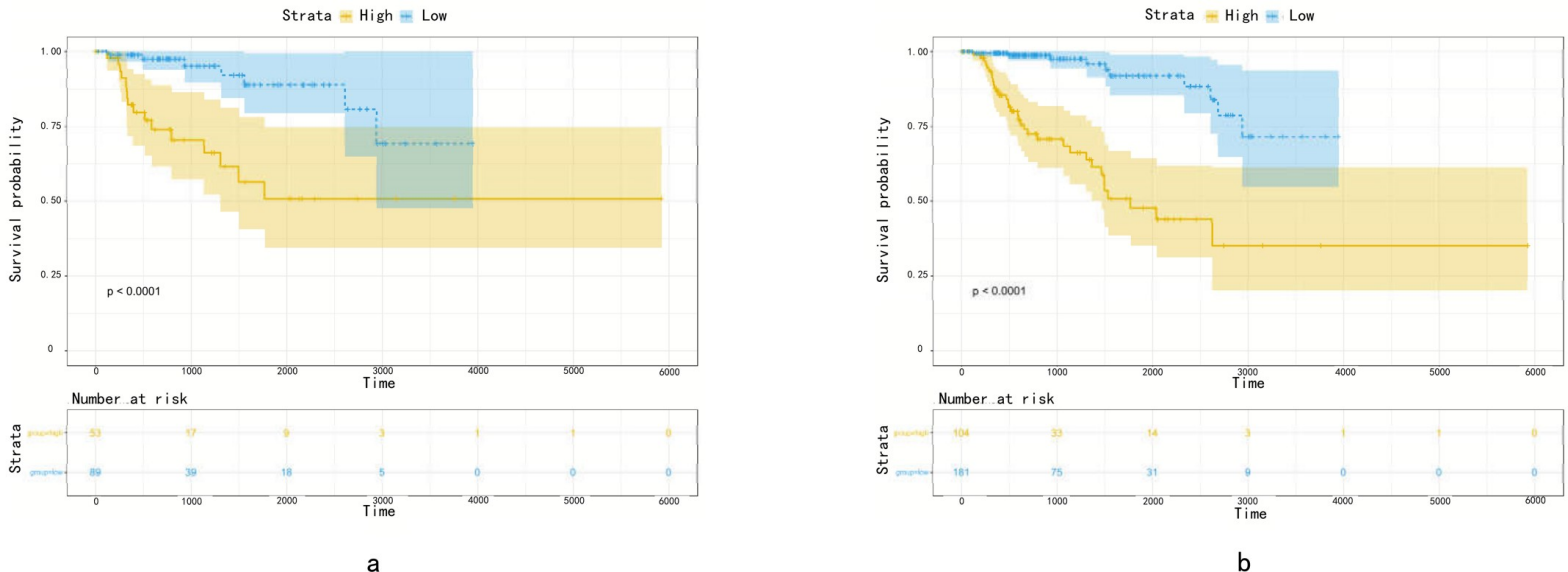
(a)Method Kaplan Meier. Kaplan-Meier estimated the survival time of the testing set patients using a risk score based on 5 mRNA signature. (b) Method Kaplan Meier. Kaplan-Meier estimated the survival time of the complete set patients from using a risk score based on 5 mRNA signature. The plot was used to visualize the survival probability for the low-risk versus high-risk group of patients based on the optimal cut-off point.

**Fig 7:**
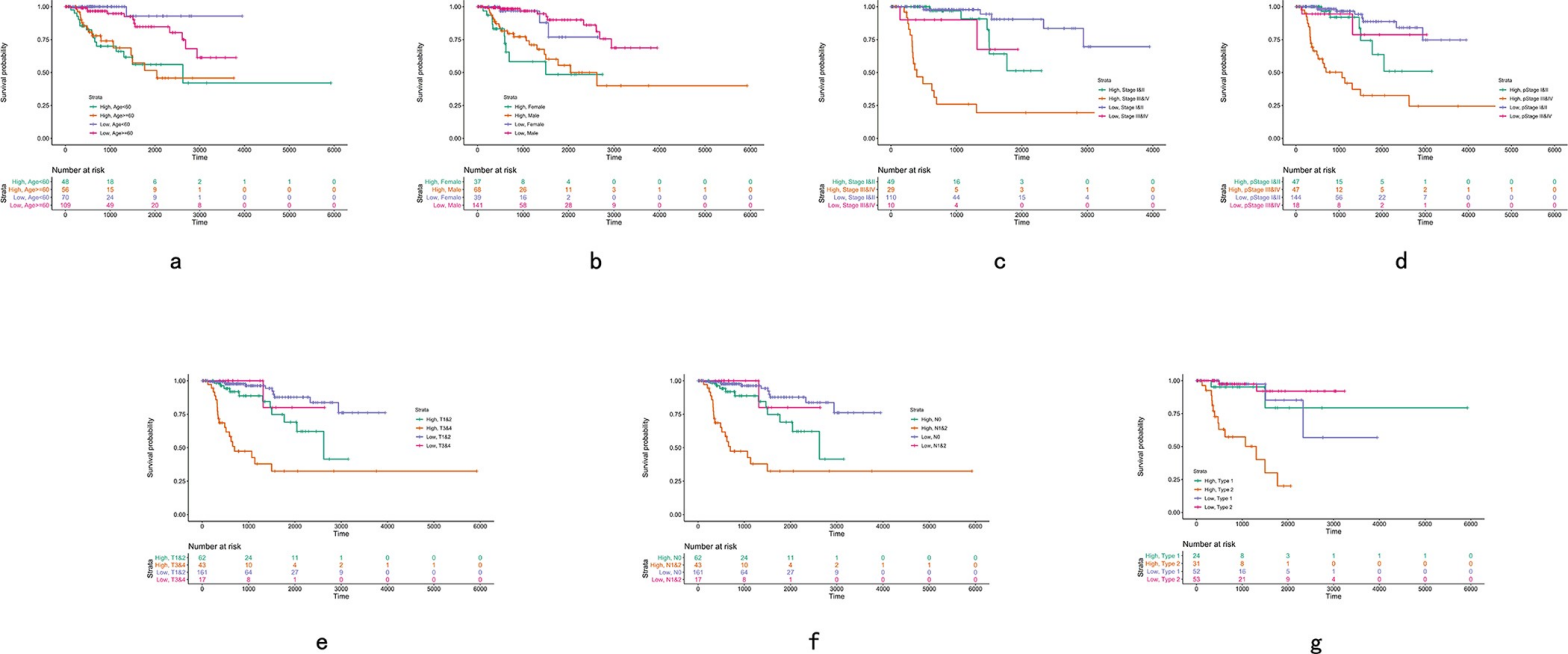
Stratified Cox hazard analysis of five gene signature on clinicopathological characteristics in pRCC cohort.

**Fig 8:**
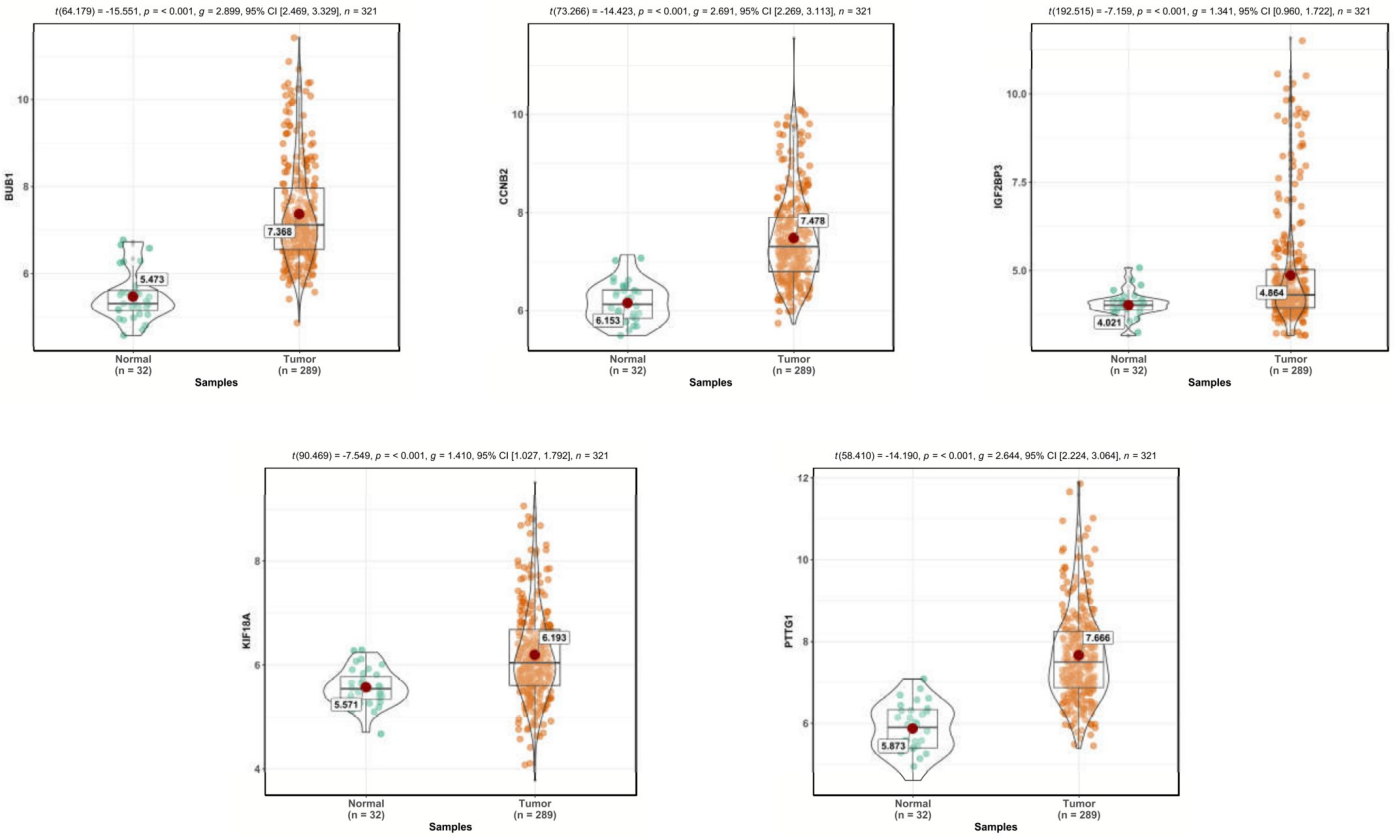
The comparative gene expression level of CCNB2, IGF2BP3, KIF18A, PTTG1, and BUB1 in normal tissue and pRCC tissue from TCGA database, respectively.

The predictive efficiency of the five-mRNA signature in testing set with 142 patients was then evaluated. The patients in the testing set were classified into high-risk (n = 53) and low-risk groups (n = 89) by using the same model and criteria. Similar to the training set, overall survival was significantly lower in the high-risk group than in the low-risk group (p < 0.0001)(Fig 6A). Risk score-based classification of the external complete set also yielded similar results as shown in Fig 6B. Despite unbalanced samples in each group, the results of survival analysis were similar to those of low-risk patients with significantly longer survival times than those with high-risk patients (*p* < 0.0001). The clinical characteristics available from the TCGA database were integrated and univariate Cox regression analysis was performed to detect the candidate clinical prognostic parameters. To validate independent predictive power of five-gene signature from clinicopathological factors in pRCC cohort such as age, pathological stage, clinical stage, pathological type and so on, the stratified Cox proportional hazard analysis was constructed and visualized in Fig 7 and Fig 8, which suggested this signature could distinguish the high-risk subgroup from low-risk one in each clinical subtype and further convinced the independent prognostic efficacy of the final gene signature.

### 3.6 Functional and pathway enrichment analysis of DEGs

**Fig 9:**
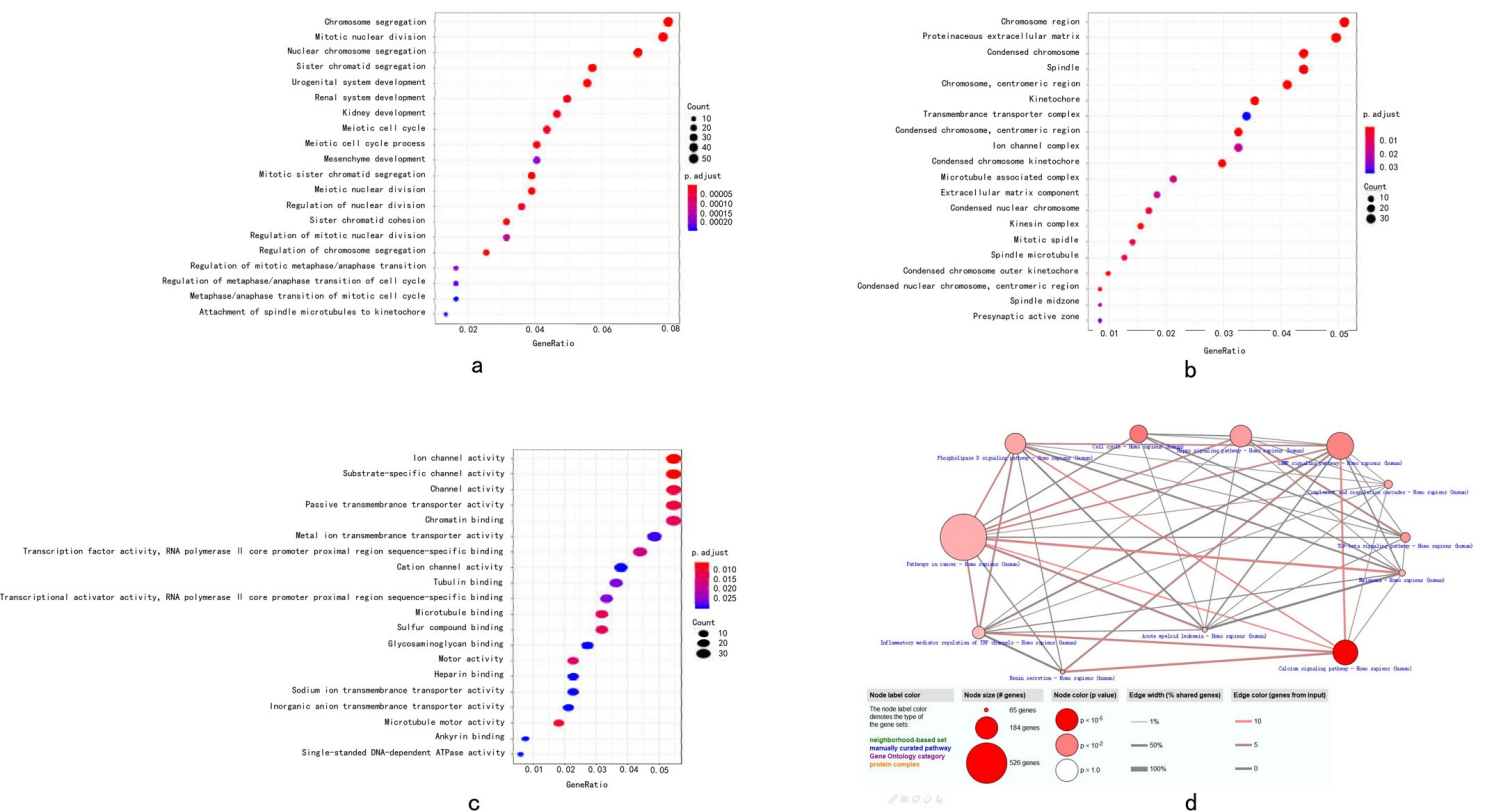
(a) biological process group of GO analysis. (b) cellular component group of GO analysis. (c) molecular function group of GO analysis.(d) The KEGG analysis. Biological function and KEGG pathway analysis of target genes. The overlapping target genes were predicted using the R language. (a-c) The enriched GO biological processes of target genes. (d) The enriched KEGG pathways of target genes.

After performing GO analysis of DEGs with clusterProfiler package in R language, the DEGs were classified into three groups: molecular function group, biological process group and cellular component group. The biological results revealed that DEGs were primarily enriched in chromosome segregation, mitotic nuclear division, nuclear chromosome segregation, urogenital system development, renal system development and kidney development. The cellular component results indicated that DEGs were mainly riched in chromosomal region, proteinaceous extracellular matrix, condensed chromosome, spindle, chromosome and centromeric region. The molecular function results showed that DEGs were mainly enriched in ion channel activity and substrate-specific channel activity (Fig 9A–9C). To investigate pathway enrichment, KEGG signaling pathway analysis was used to identify the pathways, including cAMP signaling pathway, phospholipase D signaling pathway, Hippo signaling pathway, cell cycle, complement and coagulation cascades, TGF-beta signaling pathway, inflammatory mediator regulation of TRP channels and melanoma (Fig 9D).

## Discussion

In the present study, the R language was used to analysis the gene data downloaded from TCGA databases. The total of 4553 DEGs in pRCC compared with control samples were identified, which included 2632 up-regulated and 1921 down-regulated genes. The DEGs were mainly enriched in 60 GO terms, including ion channel activity, chromosome segregation and chromosomal region. The KEGG pathway enrichment analysis result showed that the DEGs were associated with calcium,cAMP,phosphollipase D, and hippo signaling pathway. The Ca2+-mediated signaling pathways have been implicated either directly or indirectly related to tumorigenesis and tumor progression[19–21]. In this pathway, Orai1 exaggerates cell proliferation, migration, invasion and evasion of apoptosis[19]. The cAMP plays a complex and context-dependent role in regulating cell migration[22]. Usually the focus has been mainly on PKA-mediated migratory effects. Shaikh et al. suggests that hypoxia enhances cAMP-dependent protein kinase activity by up-regulating PKA gene expression in a HIF dependent mechanism and that PKA plays a key role in hypoxia-mediated EMT, migration, and invasion in lung cancer cells[23]. There is increasing evidence that the Hippo pathway is dysregulated in many human cancers, and dysregulation of the Hippo pathway exerts a significant impact on cancer development, including liver, breast, lung, colon, ovary, and others[24]. Aberrant phospholipase D (PLD) expression has been identified in multiple facets of complex pathological states, including cancer and inflammatory diseases. PLD contributes to various mitogenic or oncogenic signaling pathways[25]. Therefore, monitoring of these signaling pathway maybe beneficial to understanding the mechanism of carcinogenesis and researching treatment of prostate cancer.

We identified 5 mRNAs that are associated with the survival of patients with papillary renal cell carcinoma,namely CCNB2, IGF2BP3, KIF18A, PTTG1, and BUB1 in the training set. The prognostic related mRNAs were further selected to construct a risk score formula by Cox regression model. Besides, we used the ROC analysis to identify the optimal cutoff point, and divided patient into high-risk and low-risk groups. In the univariate Cox regression model, the survival time of high-risk patients was significantly shortened. Subsequently, the GO and KEGG pathway analysis suggest that mRNA plays a crucial role in molecular pathogenesis and progression of pRCC.

Among the 5 mRNAs which are associated with the prognosis of pRCC, some have been reported to express in cancer or other diseases, but have not been examined in pRCC. For example, elevated cytoplasmic CCNB2 protein levels are strongly associated with short-term disease-specific survival of breast cancer patients[26]. Cyclin B2 (CCNB2), the member of cyclin family proteins, regulates the activities of cyclin dependent kinases (CDKs) and different cyclins function spatially and temporally in specific phases of the cell cycle[27]. CCNB2 commonly triggers the G2/M transfer by activating CDK1 kinase[28, 29]. CCNB2 is generally located in the Golgi apparatus during both interphase and mitosis[30]. Many evidences suggested that CCNB2 expression was increased in a variety of human cancers, such as non-small cell lung cancer[31], breast carcinoma[26], gastric cancer[32], colorectal adenocarcinoma[33], pituitary adenoma[34] and adrenocortical carcinoma[35]. Therefore, CCNB2 is important in pRCC and may be used as a prognostic indicator. It has been reported that insulin-like growth factor 2 mRNA binding protein 3 (IGF2BP3) expression correlates with malignancy[36]. Schaeffer et al. suggested that IGF2BP3 was found to be selectively overexpressed in pancreatic ductal adenocarcinoma tissues but not in benign pancreatic tissues[37]. In ovarian cancer, high expression of IGF2BP3 was associated with poor survival, and women diagnosed at advanced stages with elevated IGF2BP3 was at higher risk of developing chemoresistance[38]. Lochhead et al. revealed that normal colorectal epithelium was negative for IGF2BP3 in patients of normal mucosa adjacent to carcinoma, and IGF2BP3 was associated with poor differentiation, stage III–IV disease, BRAF mutation, and LINE-1 hypomethylation[39]. Lin et al. demonstrated that IGF2BP3 enhances cell invasion ability and tumorigenicity in human OSCC in vitro and in vivo[40]. In the present study, it was demonstrated that the pRCC patients with IGF2BP3 alterations exhibited a poorer survival rate compared with those without the genetic alterations. This result suggested that the mutation in IGF2BP3 reduces the survival rate of patients with pRCC. Kif18A, a member of the kinesin super family of molecular motor proteins, regulates chromosome congregation and suppresses kinetochore movements to control mitotic chromosome alignment in the pre-anaphase state of the mammalian cell cycle[41]. Notably, Nagahara et al. demonstrated that colorectal cancer cells transfected with Kif18A cDNA demonstrated significant enhanced migration and invasion compared to mock-transfected cells[42]. KIF18A might be a biomarker for HCC diagnosis and an independent predictor of DFS and OS after surgical resection[43]. The results of the present study were consistent with the results of previous studies. KIF18A may have an important role in progression of pRCC, however this requires further study, in order to verify the specific molecular marker role of KIF18A in the diagnosis of patients with pRCC. Pituitary tumour transforming gene 1 (PTTG1) is over-expressed in a vast array of malignancies including pituitary [44, 45], thyroid[46], colorectal[47] and lung[48] cancer. The high level of PTTG1 is commonly associated with an enhanced proliferative capacity, increased tumour grade and high invasive potential. Current evidences regarding the biological role of BUB1 in cancer is contradictory. Mutations in BUB1, some of which are functional, occur in some cancers, including those that originate in the lung, colon, and are reported to be associated with chromosomal instability and lymph node metastasis, suggesting that silencing of this kinase may mediate aggressive clinical behavior[49]. Moreover, Aurora b hyperactivation caused by overexpression of BUB1 leads to misaggregation of chromosomes, thereby inducing aneuploidy[50].

## Conclusion

Taken all together, by performing a comprehensive analysis for differentially expressed mRNA profiles and corresponding clinical information, our study demonstrated that five-mRNA signature was a potential diagnostic marker in pRCC, and was an independent prognostic factor in pRCC patients. This signature has a lot of potential prognostic and therapeutic implications for the pRCC patient management. However, further research is needed to validate our findings and establish molecular mechanisms for mRNAs interactions and papillary renal cell carcinoma progression.

## Supporting information

S1 File Details of the result of univariable cox regression analysis. (XLSX)

S2 File The mRNA with p-value less than 0.05 in univariable cox regression analysis. (XLS)
